# Identification and characterization of areas of high and low risk for asymptomatic malaria infections at sub-village level in Ratanakiri, Cambodia

**DOI:** 10.1186/s12936-017-2169-1

**Published:** 2018-01-15

**Authors:** Lies Durnez, Myrthe Pareyn, Vanna Mean, Saorin Kim, Nimol Khim, Didier Menard, Marc Coosemans, Tho Sochantha, Vincent Sluydts

**Affiliations:** 1 Institute of Tropical Medicine, Antwerp, Belgium; 2 National Center for Parasitology, Entomology and Malaria Control, Phnom Penh, Cambodia; 3 Institut Pasteur du Cambodge, Phnom Penh, Cambodia; 4 University of Antwerp, Antwerp, Belgium

**Keywords:** Malaria, Hotspots, Asymptomatic carriers, Risk factors, Clusters

## Abstract

**Background:**

Malaria elimination needs a concentration of activities towards identification of residual transmission foci and intensification of efforts to eliminate the last few infections, located in so-called ‘malaria hotspots’. Previous work on characterizing malaria transmission hotspots has mainly focused on falciparum malaria and especially on symptomatic cases, while the malaria reservoir is expected to be mainly concentrated in the asymptomatic human population when transmission is low. For *Plasmodium vivax*, there has been less effort in identifying transmission hotspots. The main aim of this study was to uncover micro-epidemiological mechanisms of clustering of malaria infections at a sub-village level, based on geographical or behavioural features.

**Methods:**

A cross-sectional survey was performed in three villages within the highest malaria endemic province of Cambodia. The survey took place in the dry season, when the malaria reservoir is expected to be low and residing in the asymptomatic part of the population. Village and field locations of households were georeferenced, blood samples were taken from as many residents as possible and a short questionnaire probing for individual risk factors was taken. Asymptomatic malaria carriers were detected by PCR, and geographical clustering analysis (SaTScan) as well as risk factor analysis were performed.

**Results:**

A total of 1540 out of 1792 (86%) individuals were sampled. Plasmodial DNA was detected in 129 individuals (8.4%). *P. vivax* was most prevalent (5.5%) followed by *Plasmodium malariae* (2.1%) and *Plasmodium falciparum* (1.6%). Mixed infection occurred in 12 individuals. In two out of three villages geographical clustering of high and low malaria infection risk was clearly present. Cluster location and risk factors associated with the infection differed between the parasite species. Age was an important risk factor for the combined Plasmodium infections, while watching television at evenings was associated with increased odds of *P. vivax* infections [OR (CI): 1.86 (0.95–3.64)] and bed net use was associated with reduced odds of *P. falciparum* infections [OR (CI): 0.25 (0.077–0.80)].

**Conclusions:**

Clusters of malaria carriers were malaria species specific and often located remotely, outside village centres. As such, at micro-epidemiological level, malaria is not a single disease. Further unravelling the micro-epidemiology of malaria can enable programme managers to define the interventions likely to contribute to halt transmission in a particular hotspot location.

**Electronic supplementary material:**

The online version of this article (10.1186/s12936-017-2169-1) contains supplementary material, which is available to authorized users.

## Background

Although malaria is often considered as a single disease, human malaria is mainly caused by four species of plasmodial parasites, of which *Plasmodium falciparum* and *Plasmodium vivax* are the most common. These two species co-exist in many parts of the world including Southeast Asia. Whereas the understanding of the epidemiology of *P. falciparum* infection and disease is very broad, for *P. vivax* it is relatively poor [1].

Despite major attempts over the past century to control malaria, it remains the most important human parasitic disease worldwide, posing a health risk to more than half the world’s population [2]. The Roll Back Malaria strategy of ‘Scaling Up for Impact through universal coverage with effective interventions’, supported by an increase in malaria funding, has achieved low rates of malaria transmission in several areas and consequently a much reduced disease burden, at least for falciparum malaria [3]. Controlled low-endemic malaria can be accomplished by using prevention and treatment measures in addition to a universal net coverage. Elimination, however, requires additional activities. Intensive active and passive case detection methods need to be combined and likely a parasite specific approach will be necessary to reinforce existing control measures [3].

To interrupt with the endemic transmission, it is important to concentrate activities towards identification of residual transmission foci [4] and intensification of efforts to eliminate the last few infections [3], located in so-called ‘malaria hotspots’ [5]. Hotspots are particular areas within a focus that maintain a higher transmission intensity in comparison with the average transmission in that focus [6]. When these malaria hotspots persist over time, they can act as a recurrent source from which infection can spread to temporary hotspots. A longitudinal study in Kenya has shown that hotspots of febrile falciparum malaria are unstable whereas hotspots of asymptomatic parasitaemia were stable for several years [7]. In Sudan it was demonstrated that low-density asymptomatic infections perpetuate transmission over the dry season [8]. Other studies in Africa have shown that decreasing distances to the nearest infected person increase the risk of malaria disease (e.g. [9, 10]), showing the importance of the human reservoir and its interactions with the vector and its ecological context. It is assumed that there will be no more malaria transmission in unstable hotspots when stable infection sources are eliminated [6]. These stable malaria hotspots are characterized by the persisting occurrence of asymptomatic malaria infections. The evolutionary advantage to the parasite of sustaining chronic, low-density infections is clear, particularly since asymptomatic individuals do not seek treatment, and represent a parasite reservoir from which malaria vectors can become infected [11, 12].

Previous work on characterizing malaria transmission hotspots has mainly focused on falciparum malaria and especially on symptomatic cases (reviewed in [6]). It has been hypothesized that in low-endemic settings, mainly in a sub-Saharan Africa context, individuals with sub-microscopic malaria are the source of 20–50% of all human-to-mosquito transmission [13]. It is yet unclear whether the same holds in a Southeast Asian context as recent experiments demonstrated patients in Cambodia with smear-detectable *P. falciparum* gametocytes to be over 20 times more likely to infect mosquitoes than those without [14]. Note however that these experiments were performed on adults, whereas under 18 year olds, which are a large part of the reservoir, were not tested. Therefore, the question remains whether symptomatic or asymptomatic cases are the main reservoir of the *P. falciparum* parasite in a low-endemic Southeast Asian malaria setting. For *P. vivax*, there has been less effort in identifying transmission hotspots, and as far as we know, all but one [15] spatial and temporal analyses have been restricted to symptomatic or microscopically confirmed cases [16–25]. Spatial heterogeneity of symptomatic vivax malaria has been linked to environmental disturbances [16, 18], distance to breeding sites [16, 17], soil occupation [19, 26], or forest coverage [18].

In regions approaching pre-elimination in Southeast Asia, it has indeed been shown that the majority of malaria infections are asymptomatic [27, 28]. The epidemiology of malaria in this region is highly complex [29], with all four species of human plasmodia occurring, and this in combination with a high diversity of potential vector species [30–34]. The relative importance of vivax malaria is increasing region-wide, especially in areas where control efforts have a significant impact on falciparum malaria [18]. Moreover, the rise of artemisinin resistant falciparum malaria in the region is of global concern [35, 36], as such urging the elimination of malaria from Southeast Asia. Malaria transmission is largely restricted to forested areas, as the habitats of the main vectors, *Anopheles dirus* and *Anopheles minimus*, are closely linked to the forest [33, 37–39]. In general, risk groups for symptomatic malaria in this environment are described to be mostly forestry workers, new settlers and mobile/migrant populations who have come into forested areas, being the current target of malaria control programmes [29, 40]. Recent spatial analysis on the village-based PCR prevalence data of a cross-sectional survey shows that geographical clusters of asymptomatic malaria infection with a radius between 25 and 30 km exist in Ratanakiri, the most endemic province of Cambodia [28]. Interestingly, this study has observed that the geographical locations of these clusters are different for the different human malaria species, suggesting a different eco-epidemiology for each malaria species.

The main aim of this study was to refine this analysis to sub-village level to uncover micro-epidemiological mechanisms of clustering of malaria infections, based on geographical or behavioural features. A cross-sectional survey was performed in three villages in Ratanakiri province in the dry season, when the malaria reservoir is shown to be at its minimum [15]. Asymptomatic malaria carriers were detected by PCR, and geographical clustering analysis as well as risk factor analysis were performed.

## Methods

### Study sites and study design

The study sites are located in Ratanakiri, one of the highest endemic provinces in Cambodia, where malaria incidence counted 21 infections per 1000 persons at risk in 2012. Malaria transmission is perennial, but peaks in the wet season (June–October). Three villages (Chamkar Sann, Phi and Tun) were selected out of study villages included in the MalaResT trial [41] based on previously recorded data. They were chosen because they presented a high prevalence of infection and a low incidence of disease, from which it was deducted that there would be a higher chance that asymptomatic *Plasmodium* carriers would be found. Whereas Phi and Tun are highly forested, this is less the case for Chamkar Sann. Chamkar Sann is a village located in the south of the province. Village houses are mainly clustered along the road, and field houses are within a radius of 3 km from the village centre. Phi is located in the east of the province, at the border with Vietnam. Village houses are situated at both sides of the Tonle San river. Most field houses are based 8 km away from the village centre, in a densely forested area named ‘red soil area’, or along the road towards it. The third village, Tun, is located more in the north of the province. The village houses are situated along the road (approximately 1 km). Field houses are located further away at the northern extension of the road, or on a difficult-to-access hill close to the village centre. Farming activities in the three villages are mainly dedicated to cassava, cashew nuts, fruits and rice. Forest activities of inhabitants include hunting and collection of wood, bamboo, plants and fruits.

Between 20 January and 15 February 2016, a census team and a blood collection team visited the selected villages. The aims were updating the available population census from the previous project, taking GPS coordinates of all residences of the households, collecting blood samples from all inhabitants, and taking a short questionnaire probing for individual risk factors. Demographic variables included gender, age, main occupation and relation to the head of the household. Spatial coordinates were taken for village and field residences of most households. Blood samples were collected by finger prick and immediately stored in labeled 96-well plates and on filter papers as previously described [28, 42]. Each participant and household was provided with a unique code. As in the previous study [28], axillary temperature was measured, and participants were asked whether they had fever in the past 48 h. Individuals with fever or other malaria related symptoms were offered the possibility to take a rapid diagnostic test [CareStart™ Malaria, pLDH/HRP2 COMBO (PAN/Pf)]. Malaria positive cases were treated by dihydroartemisinin plus piperaquine combination according to the national treatment guidelines.

Behaviour-related to previous malaria exposure, self-reported bed net use, owning a plot hut and performing activities related to malaria exposure (i.e. working in the forest or plantation, watching television in the evening) was also queried (see Additional file 1: Table S1).

### Malaria PCR detection

Malaria PCR detection was performed as previously described [28, 43]. Briefly, blood samples were transported in cooled boxes by a taxi service to Institut Pasteur in Phnom Penh within 24 h. A high throughput DNA purification step was performed, and molecular detection and identification of the *Plasmodium* parasites were respectively performed by a screening real-time PCR (infections detected from 10 parasites/µl) and a nested real-time PCR on the extracted DNA in a 96-well plate format [43]. All positive samples from the screening real-time PCR were tested by a nested real-time PCR assay for *Plasmodium* species identification by using species-specific primers for the four main human malaria species (*P. falciparum*, *P. vivax*/*Plasmodium knowlesi*, *Plasmodium ovale*, *Plasmodium malariae*).

### Statistical analysis

#### Data entry

All data were collected on standardized census and blood collection forms, which were daily entered in a pre-programmed Access database. In order to reduce entry-errors, data were double entered, cross-checked and corrected. Census data, survey data and PCR data were entered in separate databases, which were merged based on a 10 digits unique code for each participant.

#### Spatial statistics

Geographical clusters with high and low risk of asymptomatic malaria infection were detected by using the spatial scan statistical software SaTScan (version 9.4.2 64-bit). Analyses were performed for all *Plasmodium* species, or for *P. falciparum*, *P. vivax* and *P. malariae* separately, and for participants’ main residence, field residence or village residence separately. Individuals without georeferenced field- or village residence were excluded from the respective analysis. Clusters were detected by gradually scanning circular windows across space (with a maximum window size of 30% of the population at risk), noting the observed and expected number of infections inside and outside each circular window. In SaTScan, the null hypothesis states that infections are randomly distributed. Isotonic clusters were assessed based on 9999 Monte Carlo simulations, and the window presenting the maximum likelihood was the most likely cluster. SaTScan reported a p value for all obtained clusters, as well as a relative risk which is the estimated risk within the cluster divided by the estimated risk outside the cluster [44].

#### Risk factor analysis

The association of individuals’ characteristics or potential risk behavior with the malaria prevalence was analysed as previously described [28] by fitting generalized linear mixed models (GLMM) with a binomial error distribution on the presence/absence of infection data by using the lme4 package [45] in R version 3.3.2 [28]. Potential bias, through clustering of individuals with malaria infections at household level, were taken into account by including the households as a random effect in the model. p values were calculated by an analysis of variance (ANOVA) test and odds ratios (OR) and its 95% confidence intervals (CI) were computed as the exponent of the model coefficients [25, 28]. All explanatory variables (village, gender, age groups, axillary temperature, past malaria, use of bed nets or hammock nets, owning a plot hut, watching television and household size) were first tested in a univariable model with individual PCR results as outcome variable. Models were constructed for all *Plasmodium* species together, as well as for *P. falciparum*, *P. vivax* and *P. malariae* separately. All variables showing a potential association (p < 0.1) in the univariable models were included in the respective multivariable random effects logistic regression models. Collinearity between variables was checked by Variance Inflation Factors (VIF) using the ‘Mass’ and ‘car’ packages in R version 3.3.2 [28]. The Akaike Information Criterion (AIC) was used for stepwise model selection using the step() function [25, 28]. The preferred model was the one presenting the lowest AIC [46]. For each category in this model, OR and 95% CI were calculated compared to the reference category.

## Results

### Summary statistics

A total of 1792 inhabitants in 344 households were registered in the population census (Table 1). Of these, 1540 (85.9%) individuals were sampled. Main reasons for not being sampled were absence from the village (e.g. due to studies, work or visiting relatives) or fear of the sampling process (finger prick). Age distribution, gender and occupational patterns were comparable between villages and between sampled versus non-sampled individuals (see Additional file 1: Table S2).

**Table 1:** Population summary statistics (2016) and PCR prevalence of malaria in Chamkar Sann, Phi and Tun

Village	No. inhabitants (households) in census	No. sampled individuals (%)	PCR prevalence (no. single-species infections; no. mixed-species infections)			
			*Plasmodium* spp.	*P. falciparum*	*P. vivax*	*P. malariae*
Chamkar Sann	619 (119)	537 (86.8)	5.8% [26; 5]	1.1% [2; 4]	3.9% [18; 3]	1.9% [6; 4]
Phi	717 (123)	590 (82.3)	9.0% [50; 3]	1.5% [9; 0]	4.1% [21; 3]	3.9% [20; 3]
Tun	456 (102)	413 (90.6)	10.9% [41; 4]	2.2% [5; 4]	9.7% [36; 4]	0% [0; 0]
Total	1792 (344)	1540 (85.9)	8.4% [117; 12]	1.6% [16; 8]	5.5% [75; 10]	2.1% [26; 7]

The number of registered inhabitants and households are reported per village, including the amount of sampled individuals. PCR prevalence is reported for all *Plasmodium* species and for each of the detected species separately. *P. ovale* was not detected. The number of single-species and mixed infections are reported between square brackets

A total of 129 (8.4%) individuals tested positive for *Plasmodium* DNA, of which 85 participants (5.5%) were determined to be infected with *P. vivax*. Besides, *P. falciparum* was identified in 24 individuals (1.6%) and 33 individuals (2.1%) were infected with *P. malariae. P. malariae* was not detected in Tun and overall, no *P. ovale* infections were identified. Only one person tested positive for malaria by RDT (out of 7 individuals with an axillary temperature exceeding 38 °C that were tested by RDT) and was treated accordingly, which results in all other infections being asymptomatic malaria carriers. 12 out of 129 infections (9.3%) consisted of mixed infections, for which all possible parasite combinations were observed (see Additional file 1: Table S3).

### Spatial analysis

**Fig. 1:**
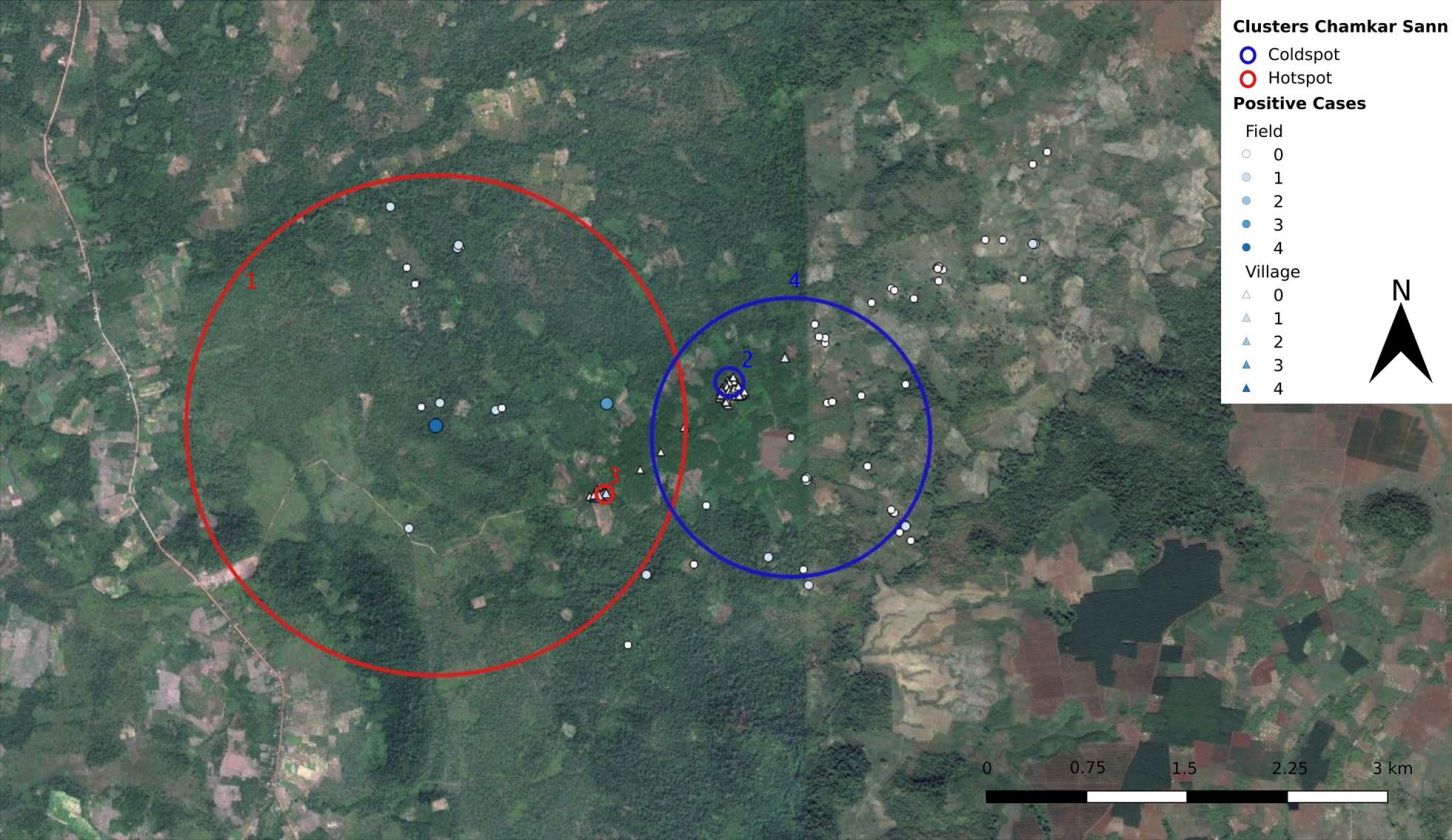
Spatial high (red lines) and low (blue lines) risk clusters for malaria infections in Chamkar Sann (2016). The amount of *Plasmodium* infections within each household is displayed by a size and colour gradient, with circles indicating field houses and triangles indicating village houses. The large and small solid red lines border two *Plasmodium* hotspots in the field, which are equivalent to the *P. vivax* high risk clusters (respectively clusters 1 and 3 in Table 2). The small solid blue line indicates a coldspot in the village centre for *Plasmodium* in general and for *P. vivax* and *P. falciparum* (Cluster 2 in Table 3), whereas the large solid blue line borders a coldspot based on the field data found for *Plasmodium* in general as well as for *P. vivax* (Cluster 4 in Table 2) (Map data: Google, Google Earth Pro)

**Fig. 2:**
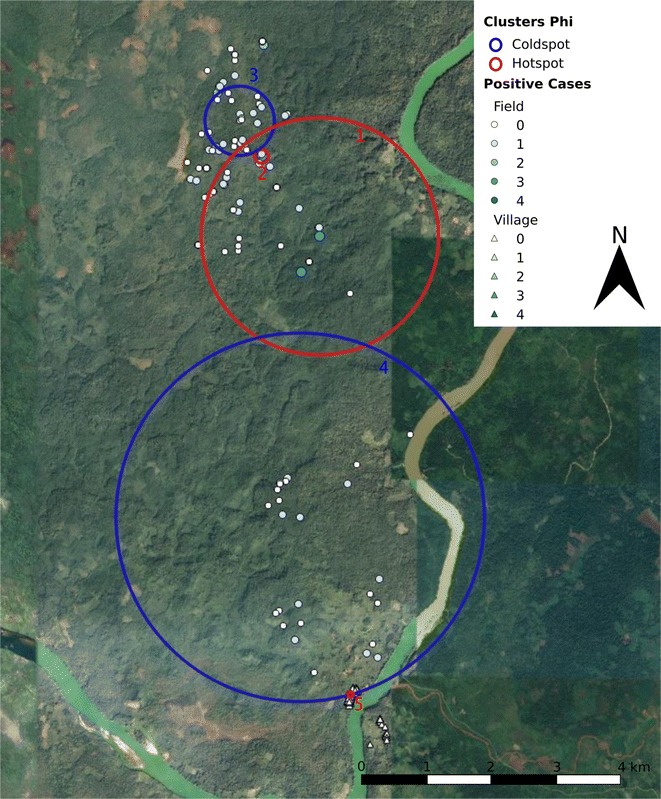
Spatial high (red lines) and low (blue lines) risk clusters for malaria infection in Phi (2016). The amount of *Plasmodium* infections within each household is displayed by a size and colour gradient, with circles indicating field houses and triangles indicating village houses. The large solid red line borders a *Plasmodium* hotspot in the field (red soil area), which is equivalent to the *P. malariae* cluster (Cluster 1 in Table 2). The upper small solid red line borders a *P. vivax* hotspot in the red soil area (Cluster 2 in Table 2), whereas the lower small solid red line borders a *P. malariae* hotspot in the village (Cluster 5 in Table 2). The large solid blue line indicates a coldspot for *P. malariae* along the road to the red soil area (Cluster 4 in Table 2). The small solid blue line borders a coldspot for *P. vivax* in the red soil area (Cluster 3 in Table 2) (Map data: Google, Google Earth Pro)

Areas of high and low relative risk of asymptomatic infections could be detected in two out of three villages (Table 2), based on the coordinates of participants’ main residence, village residence and field residence. In Chamkar Sann and Phi (Table 2; Figs. 1, 2), both clusters of high and low relative risk could be detected, whereas in Tun the distribution of asymptomatic infections was more homogeneous and only small areas of higher relative risk of asymptomatic infections were observed.

**Table 2:** Spatial high and low risk clusters of infection with *Plasmodium* parasites in general and with *P. vivax* and *P. falciparum* in Chamkar Sann, Phi and Tun (2016)

*Plasmodium*	Hot-/coldspot	X (latitude)	Y (longitude)	Radius (km)	HH	Pop	Obs	Exp	RR	p value	Cluster
CHAMKAR SANN											
MR all spp.	Hotspot	13.594419	106.971901	1.920	23	112	15	5.88	4.66	0.009	1
	Coldspot	13.597492	106.992280	0.100	24	120	0	6.30	0	0.016	2
MR Pv	Hotspot	13.594419	106.971901	1.920	23	112	10	3.62	5.70	0.009	1
	Coldspot	13.597303	106.992159	0.110	30	148	0	4.78	0	0.028	2
Village all spp.	Hotspot	13.589826	106.983550	0.064	11	67	12	4.18	4.60	0.013	3
	Coldspot	13.597303	106.992159	0.069	24	120	0	7.48	0	0.001	2
Village Pf	Coldspot	13.597303	106.992159	0.069	24	120	0	1.20	0	0.077	2
Village Pv	Hotspot	13.589801	106.983319	0.034	7	40	8	1.60	9.03	0.010	3
	Coldspot	13.597303	106.992159	0.069	24	120	0	4.79	0	0.013	2
Field all spp.	Hotspot	13.587529	106.970042	2.120	14	89	12	6.17	3.21	0.037	1
	Coldspot	13.593618	106.996415	1.070	19	88	0	6.10	0	0.006	4
Field Pv	Hotspot	13.587529	106.970042	1.800	8	53	10	2.80	7.86	0.004	1
	Coldspot	13.593618	106.996415	1.070	19	88	0	4.65	0	0.032	4
PHI											
MR all spp.	Hotspot	13.857136	107.441863	1.600	19	91	16	8.06	2.46	0.004	1
MR Pv	Hotspot	13.868187	107.433554	0.110	7	36	5	1.50	3.99	0.126	2
	Coldspot	13.873097	107.430424	0.550	20	116	0	4.82	0	0.075	3
MR Pm	Hotspot	13.857136	107.441863	1.880	27	140	12	5.32	3.93	< 0.001	1
	Coldspot	13.818306	107.439045	2.920	12	160	1	6.08	0.12	0.043	4
Village all spp.	Hotspot	13.793786	107.446222	0.043	13	63	9	5.35	1.86	0.025	5
Village Pv	Hotspot	13.794348	107.446921	0	15	80	10	3.22	5.02	0.053	6
Village Pm	Hotspot	13.793786	107.446222	0.022	10	48	5	1.66	3.80	0.001	5
Field all spp.	Hotspot	13.857136	107.441863	1.690	24	121	21	12.47	2.18	0.008	1
Field Pv	Hotspot	13.868187	107.433554	0.110	6	33	5	1.51	4	0.144	2
	Coldspot	13.873097	107.430424	0.550	27	120	0	5.48	0	0.022	3
Field Pm	Hotspot	13.857136	107.441863	1.550	18	85	11	4.06	4.28	< 0.001	1
	Coldspot	13.820585	107.436073	2.700	26	115	0	5.49	0	0.031	4
TUN											
MR all spp.	Hotspot	13.963290	107.040745	1.340	10	46	11	5.41	2.45	0.096	1
MR Pf	Hotspot	13.963290	107.040745	0	1	2	2	0.06	45.86	0.022	1
Village Pf	Hotspot	13.961924	107.053807	0	1	2	2	0.04	58.71	0.012	2
Village Pv	Hotspot	13.962842	107.053085	0.039	4	27	8	2.62	3.57	0.028	3
Field all spp.	Hotspot	13.963290	107.040745	1.820	19	82	16	10.82	1.76	0.112	1
Field Pf	Hotspot	13.963290	107.040745	0.550	5	17	4	0.33	57.41	< 0.001	1

Clusters with a p value < 0.15 are displayed

MR = only participants’ indicated main residences applied, Village = only coordinates of village houses applied; Field = only coordinates of field houses applied; all spp. = *Plasmodium* infections in general (all species); Pf = *P. falciparum*; Pv = *P. vivax*; Pm= *P. malariae*; X, Y = coordinates of centre of the cluster in longitude–latitude; HH = amount of households within the cluster; Pop = population within the cluster; Obs = amount of observed cases in the cluster; Exp = amount of expected cases in the cluster; RR = relative risk of getting infected in the cluster; Number = number assigned to the cluster, indicating clusters with (nearly) the same center point

In Chamkar Sann, *P. vivax* infections were generally clustered in the field in the western part of the area, and absence of such infections was observed mostly in the main village and in the field plots in the east. For *P. falciparum*, only an area of low risk was observed in the main village of Chamkar Sann (Table 2, Fig. 1).

In Phi, no clustering of *P. falciparum* infections was detected, whereas for *P. vivax* and *P. malariae,* areas of high and low relative risk could be identified (Table 2, Fig. 2). For both parasites, the high-risk areas are located in the ‘red soil area’ 8 km north of the main village center, but had a different geographical location. For *P. vivax*, a low risk area was also obtained in the ‘red soil area’ (more north than the high-risk area) and for *P. malariae* the low risk area was located more closely to the village. When only the village residences of the participants were taken into account, high risk areas were detected for *P. vivax* and *P. malariae*. Remarkably, the *P. vivax* cluster was situated in one house that was inhabited by 15 families (80 people) of which 10 were infected with *P. vivax*.

**Fig. 3:**
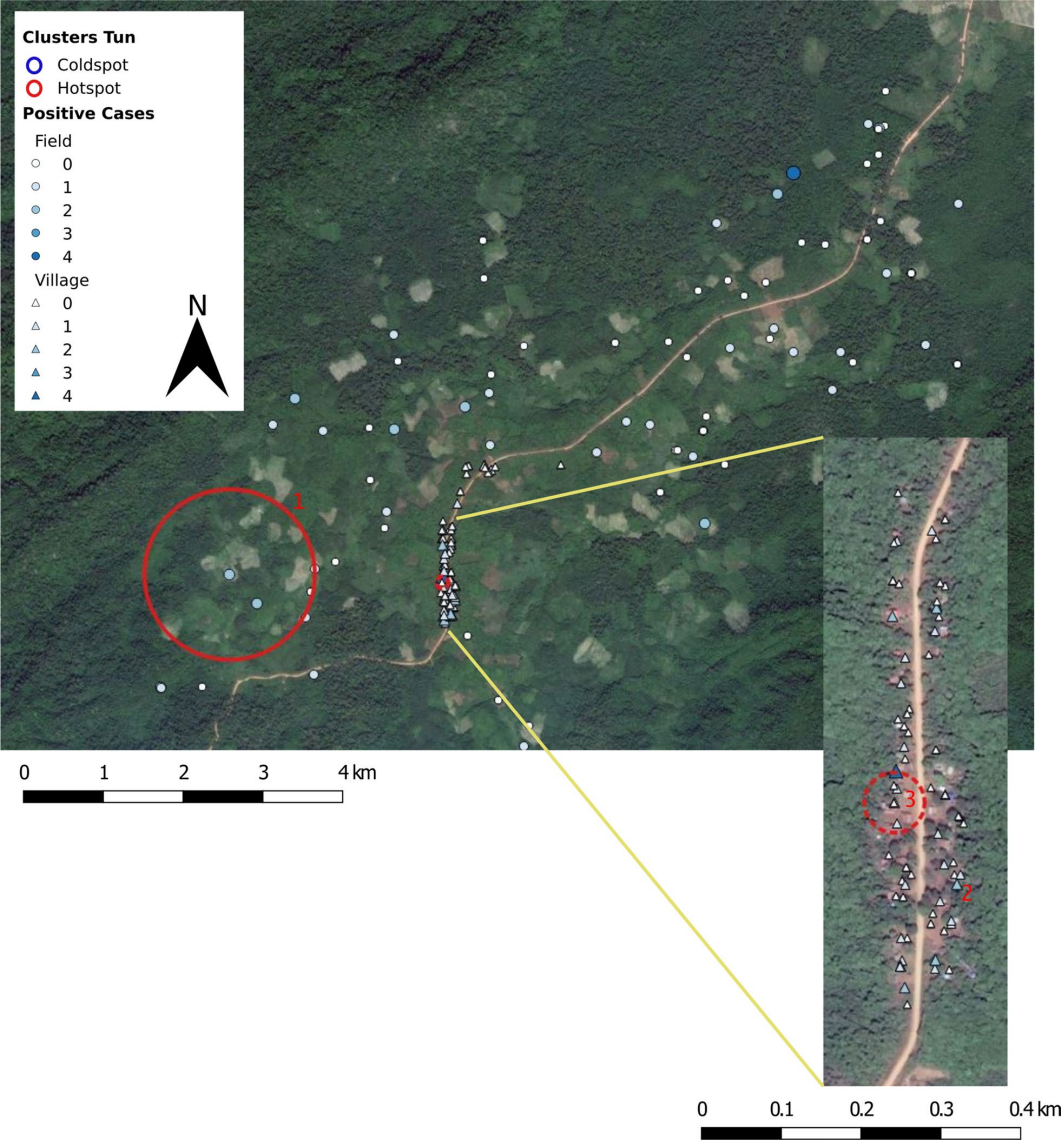
Spatial analysis of malaria infection in Tun (2016) with a zoom in on the village center (inset). Only high risk clusters were detected. The amount of *Plasmodium* infections within each household is displayed by a size and colour gradient, with circles indicating field houses and triangles indicating village houses. The solid, red line is the border of a *Plasmodium* hotspot in the field, which is nearly equivalent to the *P. falciparum* cluster (Cluster 1 in Table 2). The dashed red line indicates a *P. vivax* hotspot in the village (Cluster 3 in Table 2) (Map data: Google, Google Earth Pro)

In Tun, a few areas of high risk were detected for both *P. falciparum* and *P. vivax* (Table 2, Fig. 3). Especially when screening for species-specific clusters of high risk, radiuses were small as well as the number of households within the clusters. No areas of low risk were detected.

### Risk factor analysis

The individual risk factor models with absence or presence of malaria infection as outcome variable showed that risk factors were different for the different species. Village was an important determinant of malaria prevalence for all *Plasmodium* species combined and for *P. vivax* (Table 3). Although a higher prevalence of *P. malariae* was observed in Phi as compared to Chamkar Sann, the village parameter was not statistically significant in the multivariable *P. malariae* model. Age was a significant factor for all *Plasmodium* species combined and for *P. vivax*, with the age groups of above 6 years being mostly affected (Table 3). Ownership of a plot hut was borderline significant in the univariable analysis for *P. vivax* (p = 0.092, see Additional file 1: Table S2). It was retained in the multivariable model, but having confidence intervals overlapping the null value (OR = 1) [OR and 95% CI with No Ownership as reference: 0.543 (0.293–1.007)]. Net use significantly reduced the odds for *P. falciparum* infections [OR and 95% CI with No Net Use as reference: 0.248 (0.077–0.795)]. For *P. vivax*, net or hammock net use was not associated with the odds of infection (p = 0.885). For *P. malariae*, hammock net use was related to an increased odds of acquiring the infection [OR and 95% CI with No Net Use as reference: 6.866 (1.069–44.108)]. In the univariable analyses no differences in malaria prevalence according to gender, axillary temperature, previous malaria exposure and working activity were found (see Additional file 1: Table S4).

**Table 3:** Results of multivariable analysis comparing risk factors for infection with *Plasmodium* spp. (all species combined), *P. falciparum*, *P. vivax* and *P. malariae*

Variable	Level	*Plasmodium* spp.			*P. falciparum*			*P. vivax*			*P. malariae*		
		OR	LCL	UCL	OR	LCL	UCL	OR	LCL	UCL	OR	LCL	UCL
Village													
*NA* *=* *0*	Chamkar Sann	Reference						Reference			Reference		
	Phi	1.689	1.011	2.822				1.110	0.547	2.252	2.237	0.966	5.179
	Tun	2.084	1.223	3.552				3.006	1.525	5.927	*1	*1	*1
Age (years)													
*NA* *=* *8*	0–5	Reference						Reference			Reference		
	6–14	3.243	1.618	6.501				3.679	1.538	8.803	6.946	0.861	56.003
	≥ 15	2.342	1.210	4.533				3.116	1.251	7.763	7.203	0.944	54.937
Net use													
*NA* *=* *5*	No				Reference						Reference		
	Yes, bed net				0.248	0.077	0.795				1.133	0.234	5.479
	Yes, hammock net				*2	*2	*2				6.866	1.069	44.108
Plot hut owner													
*NA* *=* *6*	No							Reference					
	Yes							0.543	0.293	1.007			
Watch television													
*NA* *=* *6*	Never/seldom				Reference			Reference					
	Often				0.383	0.166	0.883	1.858	0.948	3.642			

Odds ratio and upper and lower 95% confidence limits were calculated with respect to the reference category, which is the first reported subgroup. For the following variables, answer categories were pooled: age (age groups 15–40, and ≥ 40); net use (treated and non-treated net use); plot hut owner (all answers stating that they have a plothut); watch television (never and seldom watching television, and often and very often watching television)

OR = Odds Ratio with respect to the reference category; LCL and UCL = lower and upper 95% confidence limits based on a total sample size of 1540 individuals from three villages; NA in variable column = number of individuals for which information concerning the specific variable was lacking

*1: No *P. malariae* cases were observed in Tun, so only Phi was compared with the reference category

*2: None of the participants reported to use a hammock net, only bed net use was compared with the reference category

## Discussion

In settings approaching malaria pre-elimination, shifts towards more heterogeneous malaria transmission are observed [6, 47]. Asymptomatic carriers within geographical clusters of high malaria transmission (hotspots) or being part of certain risk groups (hotspots) can repeatedly fuel transmission to surrounding areas or populations as the vector population expands during the wet season [6, 47, 48]. To date, this reservoir is often not (completely) covered by control strategies [49] and parasite specific approaches are non-existent [3].

In the current study, clusters of high and low malaria risks were identified in two study villages (Chamkar Sann and Phi), whereas in the third study village only clusters of high malaria risk were observed. Interestingly, when looking at different malaria species, the high-risk clusters for *P. vivax* was obtained in a different area than the *P. malariae* cluster in Phi. Such species-specific geographical clustering was also observed in a previous study carried out in 2012, in which clustering of asymptomatic malaria infections was investigated in Ratanakiri at province level [28]. Moreover, it is noteworthy that the occurrence of the specific malaria species within the current study villages are in concordance with the high risk clusters of the *Plasmodium* species detected in the previous study [28]. In particular, in 2012, Chamkar Sann was located within a *P. vivax* and a *P. malariae* cluster. Four years later, still most cases were due to *P. vivax*, and to a lesser extent to *P. malariae*. Similarly, in Phi, which was located in a high-risk *P. malariae* cluster in 2012, malaria infections were mainly caused by *P. malariae* and *P. vivax*. In Tun, situated in a high-risk *P. vivax* and a *P. falciparum* cluster, the majority of infections were due to *P. vivax* and to a lesser extent to *P. falciparum*. This spatial clustering of species implies that there might exist species-specific geographical niches, determined by various factors (e.g. behavioural, environmental or vector-related) [28], both at province and at sub-village level.

Although clusters were observed, still a considerable number of infected persons were detected outside the high-risk clusters, and transmission in Tun showed a more homogenous pattern as compared to the other two villages. This can be explained by a truly homogeneous spread of the malaria infections in Tun or due to a limitation of the SaTScan software, which scans only in circular windows [50] and does not take into account the specific shape of the village. In Tun, main residence houses are mainly located along the road, and therefore the real shape of the clusters, if existing, was possibly not similar to the circular screening windows. However, in previous micro-epidemiology studies comparing several cluster detection methods, SaTScan was the most convenient technique for the identification of geographical clusters, and is suitable for application in low prevalence settings [7, 15, 48]. No clear pattern was observed for the locations of the clusters in the different villages.

Radiuses of the geographical clusters demonstrated a consistent pattern. Areas of high and low malaria risk situated in the field (areas of low housing densities) had a relatively large radius, whereas the opposite was true for clusters in the village centre (areas of high housing densities). This might be attributed to the dispersion of malaria vectors [51]. Malaria transmission has indeed shown to be higher in the proximity of vector breeding sites, being more present in field areas [15, 52–54]. Moreover, in densely populated areas, vector flight distances remain smaller [55, 56], whereas in areas with a lower population density, female *Anopheles* mosquitoes extend their flight distance to the limits of their capacity in order to find a blood meal [57, 58], and thus can spread malaria over larger distances.

It is noteworthy that only one symptomatic case of malaria was found by RDT, indicating that the observed clusters are almost exclusively composed of asymptomatic carriers. Bejon et al. showed that groups of homesteads consisting of asymptomatic carriers can act as stable clusters over several years [7]. Therefore currently observed clusters of infection are likely to contain the parasite reservoir responsible for preserving malaria over the dry season in Ratanakiri and are thought to be responsible for recurrent transmission at the onset of the rainy season when the vector populations expand [6, 8, 47, 48]. Whether these asymptomatic cases are indeed sufficiently infectious to maintain transmission in the dry season remains to be evaluated. A follow-up study has been carried out in 2017 to verify whether the clusters are stable over time and to determine if indeed the asymptomatic cases are sufficient to maintain transmission in the dry season. A recent experiment, investigated the infectivity of falciparum gametocytaemia to *An. dirus* in Cambodia. It was demonstrated that laboratory-reared malaria vectors became mostly infectious when membrane-fed on venous blood of symptomatic parasite carriers, and only very few by feeding on blood of asymptomatic individuals [14]. While one could question the use of laboratory reared vectors, and its focus on a single vector species, *An. dirus,* while several primary and secondary vectors occur in Southeast Asia, an important drawback of the experiment is that it was conducted on the adult (18+) part of the population only. In a separate study in Thailand, it was demonstrated that only non-adult (below 18 years old) individuals became actually infectious to mosquitoes [12]. Immune responses could be different in children and adults and could have led to the results of these experiments. Therefore, further studies should be done to establish the contribution of asymptomatic carriers to transmission with respect to age for both primary and secondary vector species in Southeast Asia.

Numerous behavioural, genetic and environmental factors can be the cause of an increased risk of infection. The present study only covered certain behavioural and intrinsic individual aspects to detect a pattern among malaria-infected individuals. It is important that the described outcomes should not be over interpreted, since this study was conducted in an area where malaria prevalence was very low, although participation rate was very high in all three villages (82–91%). Shortly after this survey, a more in-depth social science study was organized in the same study sites to further extend current findings. As in a previous study in this region [15], in this study no distinction was made between new infections and relapses of *P. vivax* or recrudescences of *P. malariae*. This means that risk factors can only be interpreted as risk factors for being a *P. vivax* or *P. malariae* carrier, regardless of how long ago transmission of infection has occurred.

The current study confirms the findings of other studies in low endemic settings [15, 28, 59, 60], reporting that age is an important factor in malaria transmission, with over 6 year olds being the most important risk group. Parker et al. suggested that a yet unknown factor in the neighbourhood of schools might be the actual risk factor, since people within this age group spend most of their time at school [15]. In the present study setting however, most children work on the fields with their parents and the time that children are at school does not overlap with *Anopheles* vector biting times. A more plausible reason for this phenomenon is that the odds of getting infected are reduced when transmission is low, resulting in a shift towards an increased age of getting a first infection [59]. Additionally, when visiting the villages, it was observed that especially young people assemble outside at sunset for evening activities (e.g. playing volleyball or cards, watching television). As this aspect was only marginally explored in the current study (only ‘watching television’ was taken up in the questionnaire), outdoor evening activities require more attention and has been taken up in the follow up social science study which will be published elsewhere.

Self-reported use of bed nets was remarkably high (95%) in accordance with a previous study in Cambodia [18]. A reduction in falciparum malaria was observed among people stating to sleep under a net which is the general trend in numerous studies [18, 28]. For vivax malaria net use was not an important factor and *P. malariae* prevalence drastically increased among people using a hammock net. The latter result is likely an artefact from confounding with the location and activities associated with using a hammock net.

Forest and field activities are known risk factors for malaria, especially in Southeast Asia [28, 61]. In a recent study, more malaria infections were observed in people with temporary labour positions and plantation workers [15]. However, the current study could not confirm forest and field activities or plantation work as possible risk factor due to a very high proportion of the study participants (1460/1540) indicating to perform forest or field activities, and a very low number of study participants indicating to work as plantation worker (20/1540 on own rubber plantation; 2/1540 on someone else’s rubber plantation).

Nevertheless, in the present study most parasite carriers had their main residence in the field houses and close to the border in the village centre, adjacent to forested areas or small streams and rice fields. Other studies have indeed indicated that both proximity to the forest and vector breeding sites are significant risk factors that could elevate the malaria prevalence [18, 53, 54]. Additionally, people living in remote, hard-to-reach, forested areas are often missed by control measures [62]. In the present study a lot of effort was put in finding these people, visiting on foot or by motorbike every remote corner of the villages. Because of this effort many individuals and families, living in such remote areas were added to the original census, which was based on the bed net distribution in 2012, meaning that these individuals were not covered by this preventive measure. Other changes in the original population census were due to migration of individuals within families and between villages, districts and across the border. Therefore, migrating people, a documented risk group for malaria [63], probably still remained under-sampled despite the huge sampling effort.

**Fig. 4:**
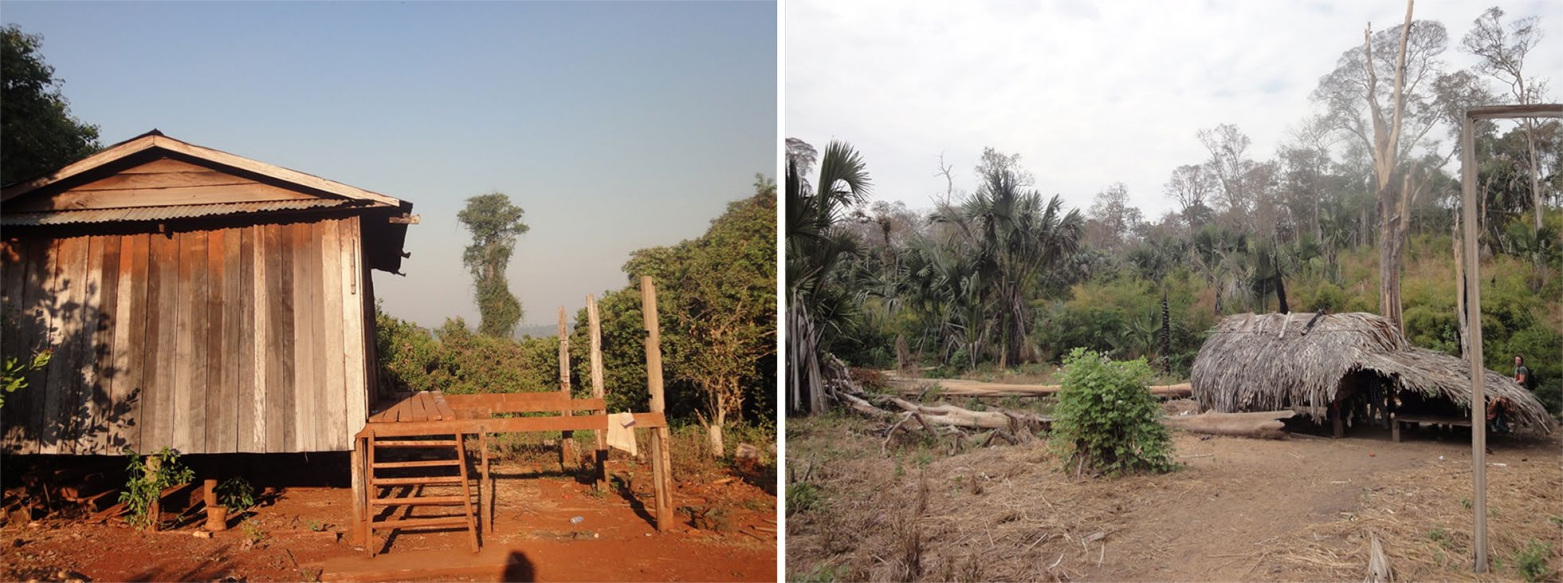
Housing structures in Chamkar Sann (2016). Left: village house, where only a minority of the cases were found; right: field house in forested area, where a lot of infections were detected

Housing structure in the field was quite poor in Chamkar Sann and Tun. Houses were open and inadequately constructed, as compared to the housing facilities in the village centre (Fig. 4). Several studies have demonstrated that improved housing structures (wood, concrete or cement with a gulf roof) are associated with a reduced risk of malaria infection [15, 60].

The impact of targeting interventions towards high risk areas or malaria hotspots remains elusive. Early results, obtained from a cluster-randomized controlled trial conducted in Kenya were unfortunately not promising [47]. However, low effective coverage, unidentified parasite carriers (e.g. due to low density of parasites), too small distance between control and intervention clusters, undersampling and undertargeting of the migrating population, the existence of parasite carriers outside the targeted hotspots, and the study running over only a single transmission season [47, 51] could have hampered the effect of the intervention. Moreover, the findings from Bousema et al. are presumably not representative for settings in Southeast Asia, since in the study site, parasite prevalence was 20.6% and different vector species occur. This suggests that validity of a hotspot targeted intervention approach should still be tested in Southeast Asia. Before conducting such a study, factor(s) that determine(s) the location of a hotspot should be identified so that interventions can be more targeted. The current study has attempted to contribute to identify such factors.

## Conclusion

In this micro-epidemiology study carried out in three structurally different villages, geographical clusters of high and low malaria infection risk were clearly identified in two villages. The geographical clustering of malaria carriers was observed to be located in remote areas, and was malaria species specific. Risk factors also differed between malaria species. As in macro-epidemiological studies, age is an important factor for vivax malaria in the current micro-epidemiology study. Net use reduced the odds for falciparum malaria. Migration and evening activities most probably are important factors and should be further investigated. As such, at micro-epidemiological level, malaria is not a single disease. In contrast to the macro-epidemiological studies, which are useful in guiding the focus of attention, further detailing the micro-epidemiology of malaria can actually enable programme managers to define the interventions likely to contribute to halt transmission in a particular hotspot location.

## Additional file

**Additional file 1: Table S1.** Survey questionnaire for collection of malariometric and risk factor data. **Table S2.** Summary characteristics from the population census of the three villages divided into the sampled and not sampled individuals for plasmodium prevalence identification. **Table S3.** Single and mixed individual plasmodium infections as detected by PCR for the three village. Pf: Plasmodium falciparum, Pv: Plasmodium vivax, Pm: Plasmodium malariae. PfPv, PfPm, PvPm and PfPvPm stands for the mixed infections occurring in one individual for multiple plasmodium species. Numbers are counts of infected individuals. **Table S4.** Results of univariate analysis comparing risk factors for infection with Plasmodium spp. (all species combined), P. falciparum, P. vivax and P. malariae. Odds ratio and upper and lower 95% confidence limits were calculated with respect to the reference category, which is the first reported subgroup.
