# Supplementary material for: Identification and characterization of areas of high and low risk for asymptomatic malaria infections at sub-village level in Ratanakiri, Cambodia
Source: Malar J. 2018 Jan 15;17:27. doi: 10.1186/s12936-017-2169-1 (PMC5769347; doi:10.1186/s12936-017-2169-1)
Supplement: Supplementary file 1 — Additional file 1: Table S1. Survey questionnaire for collection of malariometric and risk factor data. Table S2. Summary characteristics from the population census of the three villages divided into the sampled and not sampled individuals for plasmodium prevalence identification. Table S3. Single and mixed individual plasmodium infections as detected by PCR for the three village. Pf: Plasmodium falciparum, Pv: Plasmodium vivax, Pm: Plasmodium malariae. PfPv, PfPm, PvPm and PfPvPm stands for the mixed infections occurring in one individual for multiple plasmodium species. Numbers are counts of infected individuals. Table S4. Results of univariate analysis comparing risk factors for infection with Plasmodium spp. (all species combined), P. falciparum, P. vivax and P. malariae. Odds ratio and upper and lower 95% confidence limits were calculated with respect to the reference category, which is the first reported subgroup. [file 12936_2017_2169_MOESM1_ESM.docx]

Additional file 1

Additional Table S1: Survey questionnaire for collection of malariometric and risk factor data.

| **Question** | **Possible answers** |
| --- | --- |
| **Family relationship** | 1: Head  2: Spouse (= wife)  3: Daughter  4: Son  5: Grandmother  6: Grandfather  7: Other (...) |
| **Occupation** | 1: Farmer  2: Wood Logger  3: Rubber Plantation Worker  4: Civil servant  5: Uniform  6: Stay home  7: Student  8: Other (…) |
| **Age (years)** |  |
| **Gender** | 0: Male  1: Female |
| **Did you have fever the last 48 hours?** | 0: No  1: Yes |
| **Axillary temperature (°C)** |  |
| **If RDT executed, what was the result?** | 0: Negative  1: *P. falciparum*  2: *P. vivax*  3: *P. falciparum* + *P. vivax*  4: Invalid |
| **Have you had malaria in the past year?** | 0: No  1: Yes, and taken treatment from village malaria worker  2: Yes, and not taken treatment from village malaria worker |
| **Did you sleep under a mosquito net or in a mosquito hammock last night?** | 0: No  1: Yes, non-treated bednet  2: Yes, treated bednet  3: Yes, non-treated hammock net  4: Yes, treated hammock net |
| **Do you own a plothut at a farm of rice field?** | 0: No  1: Yes, only at farm  2: Yes, only at rice field  3: Yes, both one at a farm and one at a rice field  4: Yes, one plothut at farm and rice field together |
| **How many times do you watch television or dvd after sunset?** | 0: Never  1: Seldom (1-2 evenings/month)  2: Often (1-2 evenings/week)  3: Very often (>2 evenings per week) |
| **Did you in the last month…** | 0: Perform any activities in the forest (wood logging, hunting, collection of food,…)?  1: Work in your own rubber plantation?  2: Work as a worker on a rubber plantation owned by someone else?  3: Work on farms or rice fields far away from the villages?  4: Work on farms or rice fields near the village?  5: Mine for gold?  6: Other (...) |

Additional Table S2: Summary characteristics from the population census of the three villages divided into the sampled and not sampled individuals for plasmodium prevalence identification.

| Variable^*^ | SAMPLED | | | NOT SAMPLED | | |
| --- | --- | --- | --- | --- | --- | --- |
|  | Cham^§^ | Tun | Phi | Cham^§^ | Tun | Phi |
| **Gender** |  |  |  |  |  |  |
| Male | 252 | 215 | 301 | 46 | 23 | 66 |
| Female | 274 | 197 | 287 | 39 | 19 | 59 |
|  |  |  |  |  |  |  |
| **Age** |  |  |  |  |  |  |
| <5 | 90 | 59 | 97 | 8 | 10 | 21 |
| 5-15 | 274 | 239 | 299 | 52 | 28 | 72 |
| >15 | 169 | 114 | 192 | 25 | 4 | 31 |
|  |  |  |  |  |  |  |
| **Occupation** |  |  |  |  |  |  |
| Farmer | 240 | 219 | 262 | 39 | 22 | 66 |
| Wood logger | 0 | 1 | 0 | 0 | 0 | 0 |
| Rubber Plantation Worker | 3 | 0 | 2 | 1 | 0 | 0 |
| Civil Servant/Uniform | 3 | 1 | 7 | 0 | 0 | 1 |
| Student | 123 | 118 | 154 | 23 | 6 | 27 |
| Stay Home | 101 | 52 | 130 | 12 | 4 | 23 |
| Other | 29 | 21 | 33 | 1 | 9 | 8 |
|  |  |  |  |  |  |  |
|  |  |  |  |  |  |  |

^*^Missing values for different variables were omitted from this table.

^§^Chamkar Sann abbreviated as Cham.

Additional Table S3: Single and mixed individual plasmodium infections as detected by PCR for the three village. Pf: *Plasmodium falciparum*, Pv: *Plasmodium vivax*, Pm: *Plasmodium malariae*. PfPv, PfPm, PvPm and PfPvPm stands for the mixed infections occurring in one individual for multiple plasmodium species. Numbers are counts of infected individuals.

|  | Chamkar Sann | Tun | Phi |
| --- | --- | --- | --- |
| Pf | 2 | 5 | 9 |
| PfPv | 1 | 4 | - |
| Pv | 18 | 36 | 21 |
| PvPm | 1 | - | 3 |
| Pm | 6 | - | 20 |
| PfPm | 2 | - | - |
| PfPvPm | 1 | - | - |

Additional Table S4: Results of univariate analysis comparing risk factors for infection with *Plasmodium spp.* (all species combined), *P. falciparum*, *P. vivax* and *P. malariae*. Odds ratio and upper and lower 95% confidence limits were calculated with respect to the reference category, which is the first reported subgroup.

|  |  | ***Plasmodium spp.*** | |  |  | ***P. falciparum*** | |  |  | ***P. vivax*** | |  |  | ***P. malariae*** | |  |  |
| --- | --- | --- | --- | --- | --- | --- | --- | --- | --- | --- | --- | --- | --- | --- | --- | --- | --- |
| **Variable** | **Level** | **OR** | **LCL** | **UCL** | **p-value** | **OR** | **LCL** | **UCL** | **p-value** | **OR** | **LCL** | **UCL** | **p-value** | **OR** | **LCL** | **UCL** | **p-value** |
| **Village** | |  |  |  | 0.018 |  |  |  | 0.438 |  |  |  | <0.001 |  |  |  | 0.053 |
| *NA=0* | Chamkar Sann | reference | |  |  | reference |  |  |  | reference |  |  |  | reference |  |  |  |
|  | Phi | 1.693 | 1.011 | 2.835 |  | 1.362 | 0.437 | 4.246 |  | 1.127 | 0.564 | 2.254 |  | 2.163 | 0.974 | 4.806 |  |
|  | Tun | 2.102 | 1.231 | 3.589 |  | 2.086 | 0.664 | 6.548 |  | 3.001 | 1.554 | 5.794 |  | *1 | *1 | *1 |  |
| **Gender** | |  |  |  | 0.360 |  |  |  | 0.105 |  |  |  | 0.445 |  |  |  | 0.810 |
| *NA=4* | Male | reference |  |  |  | reference |  |  |  | reference |  |  |  | reference |  |  |  |
|  | Female | 0.840 | 0.579 | 1.221 |  | 0.495 | 0.206 | 1.187 |  | 0.836 | 0.527 | 1.326 |  | 1.091 | 0.537 | 2.217 |  |
| **Age(years)** | |  |  |  | 0.001 |  |  |  | 0.637 |  |  |  | 0.003 |  |  |  | 0.023 |
| *NA=8* | 0-5 | reference |  |  |  | reference |  |  |  | reference |  |  |  | reference |  |  |  |
|  | 6-14 | 3.269 | 1.631 | 6.552 |  | 1.523 | 0.364 | 6.372 |  | 3.835 | 1.609 | 9.139 |  | 8.073 | 1.018 | 64.048 |  |
|  | >=15 | 2.394 | 1.238 | 4.630 |  | 1.791 | 0.499 | 6.435 |  | 2.239 | 0.974 | 5.147 |  | 7.37 | 0.975 | 55.725 |  |
| **Axillary temp (°C)** | |  |  |  | 0.599 |  |  |  | 0.494 |  |  |  | 0.375 |  |  |  | 0.650 |
| *NA=18* | <37.5 | reference |  |  |  | reference |  |  |  | reference |  |  |  | reference |  |  |  |
|  | >=37.5 | 0.886 | 0.562 | 1.396 |  | 1.402 | 0.541 | 3.632 |  | 0.773 | 0.433 | 1.38 |  | 1.211 | 0.534 | 2.747 |  |
| **Past malaria** | |  |  |  | 0.240 |  |  |  | 0.361 |  |  |  | 0.259 |  |  |  | 0.623 |
| *NA=7* | No | reference |  |  |  | reference |  |  |  | reference |  |  |  | reference |  |  |  |
|  | Yes | 1.326 | 0.835 | 2.107 |  | 1.612 | 0.595 | 4.372 |  | 1.400 | 0.790 | 2.481 |  | 0.781 | 0.285 | 2.141 |  |
| **Net use** | |  |  |  | 0.170 |  |  |  | 0.035 |  |  |  | 0.885 |  |  |  | 0.001 |
| *NA=5* | No | reference |  |  |  | reference |  |  |  | reference |  |  |  | reference |  |  |  |
|  | Yes, bed net | 0.744 | 0.317 | 1.748 |  | 0.219 | 0.062 | 0.770 |  | 1.357 | 0.373 | 4.933 |  | 0.738 | 0.143 | 3.805 |  |
|  | Yes, hammock net | 1.781 | 0.533 | 5.954 |  | *2 | *2 | *2 |  | 1.215 | 0.174 | 8.511 |  | 8.045 | 1.157 | 55.945 |  |
| **Plothut owner** | |  |  |  | 0.291 |  |  |  | 0.550 |  |  |  | 0.092 |  |  |  | 0.795 |
| *NA=6* | No | reference |  |  |  | reference |  |  |  | reference |  |  |  | reference |  |  |  |
|  | Yes | 0.813 | 0.552 | 1.197 |  | 1.295 | 0.557 | 3.011 |  | 0.660 | 0.404 | 1.078 |  | 1.1 | 0.535 | 2.263 |  |
| **Watch television** | |  |  |  | 0.451 |  |  |  | 0.026 |  |  |  | 0.014 |  |  |  | 0.224 |
| *NA=6* | Never/seldom | reference |  |  |  | reference |  |  |  | reference |  |  |  | reference |  |  |  |
|  | Often | 1.191 | 0.752 | 1.884 |  | 0.367 | 0.157 | 0.858 |  | 2.177 | 1.122 | 4.224 |  | 0.606 | 0.272 | 1.348 |  |
| **Activities** | |  |  |  | 0.873 |  |  |  | *3 |  |  |  | 0.971 |  |  |  | 0.307 |
| *NA=11* | Forest/farm/field | reference |  |  |  | *3 |  |  |  | reference |  |  |  | reference |  |  |  |
|  | Rubber/goldmine/other | 0.930 | 0.380 | 2.274 |  | *3 | *3 | *3 |  | 1.021 | 0.336 | 3.107 |  | 2.065 | 0.563 | 7.572 |  |

OR = Odds Ratio with respect to the reference category; LCL and UCL = lower and upper 95% confidence limits based on a total sample size of 1540 individuals from three villages; NA in variable column = number of individuals for which information concerning the specific variable was lacking;

For the following variables, answer categories were pooled: Age (age groups 15-40, and ≥40); Past malaria (participants with malaria infection in the last year regardless of whether they received treatment from the village malaria worker); Net use (treated and non-treated net use); Plot hut owner (all answers stating that they have a plothut); Watch television (never and seldom watching television, and often and very often watching television); Activities (forest, farm and field activities last month, and activities in a goldmine, on a rubber plantation or other).

*1: No *P. malariae* cases were observed in Tun, so only Phi was compared with the reference category.

*2: None of the participants reported to use a hammock net, only bed net use was compared with the reference category.

*3: None of the *P. falciparum* infected participants reported to work on a rubber plantation, in a goldmine or to perform other activities. Therefore this variable could not be tested for *P. falciparum*.
